# Psycho-Vox: A Polish Questionnaire for the Differential Diagnosis of Muscle Tension Dysphonia

**DOI:** 10.3390/jcm15114145

**Published:** 2026-05-27

**Authors:** Agata Szkiełkowska, Iwona Pilchowska, Beata Miaśkiewicz, Paulina Krasnodębska

**Affiliations:** Audiology and Phoniatrics Clinic, Institute of Physiology and Pathology of Hearing, 02-042 Warsaw, Poland; a.szkielkowska@ifps.org.pl (A.S.); i.pilchowska@ifps.org.pl (I.P.); b.miaskiewicz@ifps.org.pl (B.M.)

**Keywords:** hyperfunctional voice disorders, functional voice disorders, muscle tension dysphonia, Psycho-Vox questionnaire

## Abstract

**Objective**: The aim of the study was to devise and validate a new questionnaire—Psycho-Vox—for diagnosis of muscle tension dysphonia and develop normative data for Polish-speaking adults. The tool was designed to measure 10 key psychological and social dimensions that have a bearing on functional voice disorders (FVDs), namely perseverance, coping with stress style (task-oriented or avoiding-oriented), mental well-being, resilience, burnout, family and social relationships, and tendency to project a positive self-image, tendency to exaggerate symptoms. **Methods**: The validation study involved 164 participants (46.3% were patients with muscle tension dysphonia and 53.7% were in good health; 92.7% were women). Their ages ranged from 18 to 76 years (M = 40.2; SD = 13.1). The Psycho-Vox questionnaire comprised 80 items, which participants rated on a 5-point Likert scale. Construct validity was tested using factor analysis with principal component rotation and confirmatory factor analysis. Reliability was determined using Cronbach’s *α*, and linguistic comprehension was assessed using the Flesch Reading Ease Score (FRES, Polish version) and the Jasnopis indicator. Sten norms were developed for the entire sample. **Results**: Factor analysis confirmed the 10-factor structure of the questionnaire, consistent with theoretical assumptions (KMO = 0.734; χ^2^(3160) = 8643.41; *p* < 0.001), with 89.2% classification agreement. Cronbach’s α for individual scales ranged from 0.705 to 0.837, confirming high internal reliability. The results of the FRES and Jasnopis tests indicate that the tool is understandable to adults with secondary or higher education. Significant differences were found between the patient group and the control group in terms of family relationships (*p* < 0.001), social capital (*p* = 0.003), mental well-being (*p* = 0.007), tendency to project a positive self-image (*p* = 0.004), and tendency to exaggerate symptoms (*p* = 0.005). A logistic regression model (χ^2^(10) = 62.27; *p* < 0.001; Hosmer–Lemeshow χ^2^(8) = 5.00; *p* = 0.757) showed that belonging to the patient group was positively associated with higher scores on the scales of family relationships, mental resilience, mental well-being, tendency to project a positive self-image, and symptom exaggeration, while negatively associated with perseverance and task-oriented coping with stress. The classification accuracy of the model was 73.2%. Sten norms were developed for all diagnostic scales. **Conclusions**: The Psycho-Vox questionnaire is characterised by good construct validity, high reliability, and practical clinical usefulness. The tool can be used in the differential diagnosis of muscle tension dysphonia and in monitoring the progress of treatment and rehabilitation.

## 1. Introduction

### 1.1. The Importance of Differential Diagnosis in Functional Voice Disorders (FVDs)

In recent years, the scientific literature has been addressing the issue of a growing prevalence of voice problems in the population. This applies both to professional groups who actively use their voice at work (teachers, call centre employees, actors, singers) as well as to the general adult population post-pandemic (where there have been changes in the acoustic environment, remote working, and prolonged use of the voice online) [1,2]. Psychosocial factors such as chronic stress, multitasking, and personal and organisational pressure have also contributed to the intensification of functional and psychogenic symptoms [3].

Patients with voice disorders are faced with many consequences in their daily lives. These include problems at work, reduced social functioning, poorer quality of life, but also an increased risk of recurrence of symptoms [4]. For people who use their voice professionally, there is also the difficulty of performing their job properly, which contributes to an increase in work-related stress [3]. All this contributes to the growing importance of proper differential diagnosis of patients, sometimes requiring an individualised approach or perhaps a multidisciplinary diagnostic and therapeutic team [5].

From a clinical point of view, appropriately selected psychological questionnaires not only aid in making a differential diagnosis but can also assist in monitoring the patient’s progress in therapy and rehabilitation. In this way, a questionnaire can open the way for an individual treatment plan based on how the patient is responding [6].

### 1.2. Psychological Factors in FVDs

An increasing number of scientific studies indicate that voice disorders are not only medical in nature but should be considered as part of a biopsychosocial picture. This approach assumes there are interactions between physiological factors (including phonatory efficiency of the glottis), social factors (support and social capital, burnout), and psychological factors (mental state, coping with stress, etc.). The psychological component has been marginalised for many years, but in recent years it has been recognised as a key element in the differential diagnosis of FVDs or malregulative dysphonia [7].

Understanding and controlling the psychological profile of patients with a FVD therefore becomes a priority in differential diagnosis and treatment planning. Table 1 presents the authors’ original summary of the psychological and social factors most commonly reported to affect both the diagnosis of voice disorders and the monitoring of treatment outcomes.

### 1.3. PROM as the Standard in Modern Diagnostics

According to the recommendations of both evidence-based medicine (EBM) and evidence-based assessment (EBA), standardised patient-reported outcome measures (PROMs) are important elements in making a differential diagnosis. These are usually self-report questionnaires completed by the patient which can provide important information for doctors, psychologists, or speech therapists about social and psychological factors important in making a diagnosis and in monitoring treatment. The PROMs should be properly tested for reliability and accuracy, thereby enabling standardised diagnostic procedures and uniform interpretation of results. However, the most important property is that they have been validated for the target population [14]. Such an approach is particularly important in phoniatrics, where the behavioral profile of a patient needs a tool that is tailored to the specific symptoms and mechanisms of voice disorders. In clinical practice, such a tool could be designed for use at the screening stage—for indicating a possible psychofunctional component—and then as an aid in deciding on further diagnostics (e.g., a psychological consultation) or for monitoring therapy and rehabilitation, allowing the pace and direction of apparent changes in the patient to be assessed.

### 1.4. Limitations of Psychological Tools Used to Date

In current clinical practice, it is common to use psychological questionnaires that are not specific to FVD, since there is a lack of diagnostic tools with normative values calculated for such patients. Drawing conclusions based on psychological questionnaires that do not have normative values for the target group can lead to erroneous conclusions and contribute to inappropriate therapeutic decisions. Furthermore, cross-sectional studies on PROMs have shown that even commonly used psychological questionnaires can lead to misinterpretations if they are not tailored to the clinical context [15]. From the perspective of EBA and its recommendations, the use of questionnaires not tailored to the target group is contrary to the principle of matching the diagnostic tool to the construct and the population.

Systematic reviews indicate that the PROMs currently used in voice disorders have many psychometric gaps and are of limited use in both the diagnosis and monitoring of therapeutic progress [16,17]. Thus, there is an urgent need to develop a tailored diagnostic tool for patients with voice disorders.

This paper describes how we developed a specific tool, Psycho-Vox, which combines behavioural and physiological perspectives with an assessment of psychological factors specific to patients with functional voice disorders. Such a tool needs to be constructed and reported in accordance with current standards (including content validation with the participation of experts and patients), reliability and validity analysis (including differential analysis between clinical and non-clinical groups), as well as the development of norms for the Polish adult population (so that clinicians can interpret the results in terms of clinically significant deviations) [18]. The inclusion of such a questionnaire in phoniatric practice would fulfil the requirements of EBM/EBA and optimise decision-making, minimising the risk of using poorly selected, non-standardised scales which, although popular, do not provide reliable data for this patient population [19]. In the case of FVDs, such errors may perpetuate an incorrect diagnostic pathway.

### 1.5. The Aim of the Study

This article describes how the Psycho-Vox questionnaire was developed and discusses the results of its validation. Normative values for our Polish adult group are also presented. We discuss the usefulness of the questionnaire in the differential diagnosis of muscle tension dysphonia (MTD), as well as in monitoring treatment and rehabilitation.

Validation of the Psycho-Vox questionnaire involved measuring the behavioural syndromes of the patient group. The analysis was based on comparing the target group (patients with MTD) with healthy individuals.

## 2. Materials and Methods

### 2.1. Structure of the Psycho-Vox Questionnaire

The Psycho-Vox questionnaire was developed by our interdisciplinary team—a psychologist, psychometrists, audiologists, and phoniatricians from the Audiology and Phoniatrics Clinic at the Institute of Physiology and Pathology of Hearing in Kajetany Tables S1 and S2. Psycho-Vox consists of 80 items that measure the following dimensions: first, a control scale (tendencies to project a positive self-image and exaggerate symptoms), and then levels of perseverance, ability to cope with stress (task-oriented and avoidance-oriented), social engagement (relationships with family and social involvement), resilience, burnout, and mental health. Importantly, the number of items assigned to each psychological dimension was quantitatively balanced to ensure comparable representation of all constructs within the questionnaire. The questionnaire was completed by the patients themselves. Their task was to respond to statements on a Likert scale of from 1 to 5 (1 definitely not to 5 definitely yes). The average time to complete the questionnaire was 10 to 15 min.

### 2.2. Participants

The validation study included results from 164 individuals. Although the sample size may be considered modest in relation to the number of questionnaire items, the study was designed as an initial validation and exploratory psychometric assessment of a novel clinical tool developed for a specific patient population. Recruitment was limited by the relatively narrow clinical profile of patients with muscle tension dysphonia referred to a tertiary phoniatric centre. Nevertheless, the adequacy of the sample for factor analysis was supported by satisfactory KMO values and Bartlett’s test results. Patients with MTD accounted for 46.3% of the study population, with the remainder being healthy individuals. The majority of the results were from women (92.7%), while men accounted for 7.3% of the sample. The high proportion of women is due to two factors. First, the material collected for the study included all patients presenting to the Audiology and Phoniatrics Clinic in 2025. The structure of our study population is consistent with previously published epidemiological data, which indicate a marked predominance of women among individuals with FVD. Second, a substantial proportion of patients presenting with MTD were teachers, a professional group in which women are also overrepresented. The age of the participants ranged from 18 to 76 years (M = 40.20; SD = 13.12), with a distribution close to normal. The average length of service was nearly 14 years (M = 13.9; SD = 13.2). The details are presented in Table 2, broken down into a control group (*n* = 88) and a patient group (*n* = 76).

### 2.3. Statistical Analysis

Two indicators were used to assess the linguistic comprehensibility of the questionnaire: the Flesch Reading Ease Score (FRES, Polish version) [13] and Jasnopis [20]. The FRES index determines the difficulty of a text based on sentence length and the number of syllables in words—higher values indicate that the text is easier to understand. Jasnopis, on the other hand, based on a linguistic analysis of the Polish language, classifies texts on a 7-point scale from 1 (very easy) to 7 (very difficult). The use of both measures comprehensively assesses the accessibility of the language used in a research tool.

Statistical calculations were performed using JASP and JAMOVI software. Statistical inference was based on a significance level of *p* < 0.05. Due to the exploratory nature of the study, both exploratory and confirmatory factor analysis were used. Exploratory factor analysis was performed using principal component extraction with varimax rotation. Item retention was based on theoretical consistency, factor loadings, eigenvalues greater than 1, and Velicer’s MAP criterion. Items with weak or ambiguous loadings were evaluated in relation to conceptual coherence and retained only when theoretically justified.

## 3. Results

### 3.1. Factor Validity

The factor validity of the Psycho-Vox questionnaire was verified using factor analysis with principal component rotation. The measure of fit to the data was based on a Kaiser–Meyer–Olkin (KMO) test. The results of the test were KMO = 0.734; χ^2^(3160) = 8643.41; *p* < 0.001. This result, together with Velicer’s MAP method, indicated that a 10-factor solution was optimal, similar to the structure proposed here for our Psycho-Vox questionnaire (its eigenvalue was >2 for 10 components). Classification agreement was assessed as 89.2%. Thus, we decided to verify factorial validity using confirmatory factor analysis.

The confirmatory factor analysis was aimed at empirically confirming the postulated factor structure of the Psycho-Vox questionnaire. The model was estimated using a robust estimator that took into account the categorical nature of the data. The results showed that the multi-factor model fit significantly better than the independent model, which was confirmed with a chi-square test (χ^2^(3034) = 4224.28, *p* < 0.001). The fit indices suggest that the theoretical structure was a moderately good representation of the empirical data. The RMSEA value was 0.049, and its 90% confidence interval ranged from 0.045 to 0.053, indicating a satisfactory fit of the model. The CFI, TLI, and IFI were 0.818, 0.810, and 0.821, respectively, which, given the complexity of the model and the number of items, indicates an acceptable level of fit. The SRMR value was 0.108, while the GFI reached 0.890.

Most items in the questionnaire showed statistically significant and adequately high factor loadings on their assigned latent dimensions. Within domains such as perseverance, family relationships, resilience, burnout, task stress, mental well-being, support, positive self-image, and tendency to exaggerate, the estimated loadings were generally high and stable, most often exceeding 0.60, and their confidence intervals did not include zero in the vast majority of cases.

All factors, except for stress avoidance, showed significant variance, which means that they are able to effectively differentiate between subjects. The covariance between factors was consistent with theoretical predictions, supporting the validity of the construct. Perseverance was positively associated with burnout and exaggeration tendency and negatively associated with resilience and mental well-being. Resilience correlated positively with support and overall mental well-being, and negatively with exaggeration. Burnout, in turn, was found to be associated with impaired mental well-being and lower levels of support. The pattern of relationships between the factors reflects their theoretical consistency and confirms that the questionnaire measures the constructs in accordance with the assumed concept.

The results indicate that the factor structure of the Psycho-Vox questionnaire is generally confirmed by the empirical data. The theoretical model corresponds to the structure obtained in the analysis, and most items are characterised by strong and significant factor loadings. These data support the construct validity of the tool and provide a solid basis for further psychometric analyses.

### 3.2. Reliability of Scales

High Cronbach’s alpha coefficients (0.705–0.837 in the group of patients) confirmed the listed diagnostic scales of the Psycho-Vox questionnaire. This result indicates that Psycho-Vox can be used in both scientific research and for individual diagnosis. The details are presented in Table 3. In addition, the average FRES score was in the range of 40–45 points, which indicates that the individual items are understandable to adults with secondary or higher education. In turn, the average level of clarity (approximately 4.5 on a 7-point scale) confirms that the language used in the questionnaires is accessible, although it requires basic language skills and concentration on the part of the recipient (cf. Table 3).

### 3.3. Characteristics of Diagnostic Scales

The Psycho-Vox questionnaire indicators were calculated by summing up the responses to the relevant items. Table 4 presents the basic descriptive statistics of the created variables. Analysis of the distribution measures also showed that the distribution of all created indicators is close to normal.

### 3.4. The Relationship Between Gender and Age and Diagnostic Scales

A *t*-test for independent samples showed that for most of the Psycho-Vox questionnaire indicators, there were significant differences between the groups. Subjects classified as patients obtained higher scores on the following scales: family relationships (*p* < 0.001), social capital—relationships with others (*p* < 0.01), mental well-being (*p* < 0.01), as well as having a greater tendency towards projecting a positive self-image (*p* < 0.01) and exaggerating symptoms (*p* < 0.01). In addition, patients scored somewhat higher on resilience (*p* = 0.054) and on avoiding-oriented coping with stress (*p* = 0.067). No differences were observed between the groups in terms of perseverance, burnout, and task-oriented coping with stress. The detailed results are presented in Table 5.

Pearson’s *r* correlation analysis showed that, in the control group, there was an increase in resilience, task-oriented coping with stress, and mental well-being as age and length of service increased. In addition, with both age and length of service, there was a decrease in the burnout score. However, in the patient group, no correlation was observed between any of the Psycho-Vox questionnaire indicators and age and length of service. The results are presented in Table 6.

### 3.5. Prediction of MTD

A significant logistic regression model was constructed in which each of the diagnostic indicators of the Psycho-Vox questionnaire were introduced as predictors (χ^2^(10) = 62.27; *p* < 0.001). The good fit of the model was also confirmed by the result of a Hosmer–Lemeshow test (χ^2^(8) = 5.00; *p* = 0.757). The percentage of correct classifications was found to be 73.2%. Most of the Psycho-Vox questionnaire indicators proved to be significant predictors in the model (with the exception of burnout, avoidance coping with stress, and social capital). It was observed that higher scores were associated with classification into the patient group with an increase in the assessment of family relationships (B = 0.093; *p* = 0.027), resilience (B = 0.127; *p* = 0.061), mental well-being (B = 0.179; *p* = 0.003), tendency to project a positive self-image (B = 0.191; *p* < 0.001), and tendency to exaggerate symptoms (B = 0.159; *p* < 0.001). Furthermore, with an increase in perseverance (B = −0.140; *p* = 0.014) and task-oriented coping with stress (B = −0.224; *p* < 0.001), the likelihood of classifying the subjects into the research group decreased. The detailed results are presented in Table 7.

### 3.6. Normative Values

Table 8 and Table 9 present normative values for individual diagnostic scales of the Psycho-Vox questionnaire. Sten scales were calculated due to their clinical adequacy. The sten scale (1–10) differentiates extreme results, both extremely high and low. The interpretation of sten norms is as follows:

- Sten 1–2—very low results
- Sten 3–4—low results
- Sten 4—slightly below average results
- Sten 5–6—moderate results
- Sten 7—slightly elevated results
- Sten 8—high results
- Sten 9–10—very high results.

After completing the questionnaire, the respondent’s answers were rated according to the answer key. The result obtained was then converted into stens according to the tables below. Converting the results to a sten scale allows results to be compared with each other and with how the analysed patient performed in relation to the adult population in Poland. Despite significant differences between the group of patients with MTD and the control group, it was decided to convert the general norms mainly due to the ongoing work on creating a psychological profile of patients with MTD. Another indication for converting the general norms is the fact that for most diagnostic scales, the differences obtained have a small effect size (Cohen’s d below 0.50).

## 4. Discussion

The aim of the study was to validate the Psycho-Vox questionnaire, a psychological tool developed for the interdisciplinary diagnosis of muscle tension dysphonia. The results indicate that the questionnaire meets contemporary psychometric standards, and its factor structure and reliability allow it to be used to assess psychological components relevant to voice production.

The obtained factor model is consistent with theoretical assumptions and covers the main domains of mental and social functioning of patients with voice disorders. High KMO values (0.734) and the significance of Bartlett’s test confirm the adequacy of the data for factor analysis. The convergence of the results with Velicer’s MAP method further indicates the stability of the structure. The solution obtained corresponds to the biopsychosocial concept of voice disorders, according to which psychological factors (e.g., emotional regulation, coping with stress) and social factors (relationships and support) may influence the severity and course of symptoms [3,21].

Furthermore, the Cronbach’s α coefficients obtained (0.705–0.837) confirm the high internal consistency of the tool, which allows Psycho-Vox to be treated as a diagnostic scale that meets the criteria for clinical research. The highest reliability was obtained on the scales of tendency to exaggerate symptoms (α = 0.837) and family relationships (α = 0.822). These results are similar to those obtained in other adaptations of psychological tools used in phoniatrics [22].

Interestingly, patients with MTD obtained higher scores in several domains that may initially appear counterintuitive, including mental well-being, family relationships, social capital, and resilience. These findings should be interpreted cautiously. One possible explanation is that some patients may develop compensatory coping mechanisms or receive increased social support after prolonged experience with chronic voice problems. Another explanation may involve response-related factors, including the tendency toward positive self-presentation identified in the same group. These findings are consistent with previous studies indicating a relationship between stress, burnout, emotional regulation, and the occurrence of dysphonia symptoms [3,17]. Demographic differences between groups, particularly age and professional experience, may also have contributed to these results. Patients with MTD were older and had longer work experience, which may independently influence psychological functioning, coping strategies, resilience, burnout, and social variables. Correlation analyses partly addressed this issue by examining relationships between demographic variables and Psycho-Vox scales within each group; nevertheless, future studies should include age-adjusted analyses and more demographically matched control groups.

The logistic regression model allowed us to identify factors that significantly differentiate patients from healthy individuals. Higher scores on the scales of self-image, symptom exaggeration, family relationships, and mental well-being were associated with classification into the patient group, while higher levels of perseverance and task-oriented coping with stress decreased this likelihood. These results confirm that patients with MTD are more likely to present an emotional-cognitive pattern characterised by an avoidant coping style and excessive focus on symptoms. Similar relationships have been reported in studies on the psychological determinants of functional dysphonia [18].

## 5. Limitations

Several limitations of this study should be emphasised. First, the study sample was dominated by women, which makes it difficult to generalise the results to the male population. Second, the study was cross-sectional in nature, so the temporal stability of the questionnaire was not assessed. Thirdly, cross-validation with other psychological measures was not included. In addition, the purposive sample used may have been biased by self-selection. Another important limitation is the relatively limited sample size in relation to the 80-item structure of the questionnaire. Although both exploratory and confirmatory factor analyses yielded acceptable psychometric parameters, the participant-to-item ratio remains lower than that recommended in some psychometric guidelines. Therefore, the factorial structure should be interpreted cautiously and verified in larger independent samples in future studies. Finally, the lack of comparison with clinical groups with different profiles (e.g., organic voice disorders) limits the possibility of a full assessment of the differential validity of the tool.

Despite these limitations, Psycho-Vox is the first psychometric tool in Poland developed and standardised for patients with MTD. It allows for the assessment of psychological and social factors contributing to the aetiology and course of MTD, as well as the monitoring of the effects of voice therapy. The use of Psycho-Vox in phoniatric practice is in line with the standards of evidence-based medicine (EBM) and evidence-based assessment (EBA), according to which both medical and psychosocial components should be taken into account during the diagnostic process.

Furthermore, the gap in psychometrics (the lack of other scales validated in the FVD patient population) makes the development and validation of the Psycho-Vox questionnaire a crucial step towards compliance with the principles of EBM and EBA. This questionnaire can not only streamline the diagnostic process but also improve the quality of treatment and reduce systemic costs. In this way, it would respond to urgent clinical and research needs, in line with global trends in medicine and psychometrics. Further research should include a test–retest analysis to determine the repeatability of results at different measurement points. Cross-validation in clinical groups with different voice disorder profiles would also be useful to assess the differential validity of the tool. Another important direction is to examine the impact of demographic and occupational variables, such as age, length of service, and the nature of voice strain, on the results obtained. It is also advisable to develop a shortened version of the questionnaire, adapted to outpatient practice, and to analyse the sensitivity of the tool to changes occurring during voice therapy.

The Psycho-Vox questionnaire demonstrated satisfactory validity, reliability, and linguistic comprehensibility in the assessment of psychosocial dimensions associated with muscle tension dysphonia. The tool captures multiple psychosocial dimensions relevant to patients with muscle tension dysphonia and may support interdisciplinary diagnostic and therapeutic processes in phoniatric practice. The developed normative data facilitate clinical interpretation of results in the Polish adult population. Although further validation in larger and more diverse clinical samples is necessary, Psycho-Vox represents a promising instrument for the assessment and monitoring of psychological factors associated with functional voice disorders.

## 6. Conclusions

The Psycho-Vox questionnaire demonstrated satisfactory validity, reliability, and linguistic comprehensibility in the assessment of psychosocial dimensions associated with muscle tension dysphonia. The tool captures multiple psychological and social domains relevant to patients with functional voice disorders and may support interdisciplinary diagnostic and therapeutic processes in phoniatric practice. The developed normative data facilitate interpretation of results in the Polish adult population and may support both clinical assessment and monitoring of therapy outcomes. Despite the need for further validation in larger and more diverse clinical groups, Psycho-Vox represents a promising instrument for the standardisation of psychological assessment in patients with muscle tension dysphonia, in line with the principles of evidence-based medicine and evidence-based assessment.

## Figures and Tables

**Table 1:** Summary of psychological markers in functional voice disorders (FVDs).

Psychological Factor	Significance in FVDs
Mental well-being (Polish: stan psychiczny)	It has been shown that people with FVD have a higher severity of depressive and anxiety symptoms compared to the general adult population. It has been observed that emotional regulation disorders contribute to lower quality of life reported by patients with dysphonia [8].
Perseverance (Polish: perseweratywność)	There are not many studies on the relationship between perseverance and the diagnosis of FVD. In the case of chronic symptoms, it has been found that people with high perseverance adapt less well to stressors in the workplace, which leads to chronic voice problems. It can be assumed that a fixed pattern of negative emotions and thoughts can lead to phonatory overload, which affects the treatment and rehabilitation of voice problems [9].
Coping with stress: task oriented or avoidant (Polish: radzenie sobie ze stresem: zadaniowe lub unikowe)	The dominant task-oriented style among teachers is associated with less severe symptoms of FVD, while the avoidant and emotional styles are associated with more severe dysphonia [7]. Furthermore, avoidance coping with stress reinforces subjective feelings associated with voice problems.
Resilience (Polish: odporność psychiczna)	In groups of people who use their voice professionally, it has been found that a higher level of mental resilience in people with dysphonia is associated with both fewer chronic voice problems and faster improvement in health [3].
Burnout (Polish: wypalenie zawodowe)	In teachers, more severe burnout symptoms, i.e., emotional exhaustion and depersonalization, are strongly associated with the occurrence of voice problems [10]. Furthermore, it can be assumed that patients with high levels of burnout have longer recovery times and poorer voice therapy outcomes [11].
Family relationships (Polish: czynnik społeczny: relacje z rodziną)	A review by Eadi showed that people who reported strong support from their loved ones coped better with communication limitations in FVDs and recovered their vocal abilities more quickly. Studies have shown that patients with higher levels of support, including from family, are more likely to complete voice therapy with better results [12].
Social capital—relationships with others (Polish: czynnik społeczny: kapitał społeczny)	Patients who report high social capital achieve better outcomes in the treatment of chronic diseases and cope more adaptively in health crises [13]. Other studies indicate that low social support can be both a predictor of longer recovery and a consequence of voice disorders. It can lead to a vicious circle in which dysphonia can cause limitations in professional life and social participation, which in turn reduces the sense of social support. A reduced sense of support can, in turn, increase the patient’s psychological burden and prolong recovery time [3].
Tendency to project a positive self-image (Polish: skłonność do pozytywnej autoprezentacji)	A tendency towards projecting a positive self-image may make it more likely to minimise one’s symptoms, as well as receive a positive perception by doctors and family [11]. This may lead to the problem being downplayed and the severity of dysphonia symptoms being underestimated in a diagnosis.
Tendency to exaggerate symptoms (Polish: skłonność do wyolbrzymiania symptomów)	The tendency of patients to exaggerate their symptoms is a common diagnostic problem. This may become a problem at the stage of making a differential diagnosis between functional and organic voice disorders, but it may also significantly reduce the effectiveness of treatment and rehabilitation.

**Table 2:** Characteristics of the study participants.

	Control Group (*n* = 88) (Patients Without Voice Disorders)	Patients with MTD (*n* = 76)
Percentage of women	88.6	97.4
Age, M (SD)	34.1 (10.6)	47.4 (12.2)
Years of work experience, M (SD)	8.7 (8.7)	23.2 (11.8)

M—mean; SD—standard deviation.

**Table 3:** Cronbach’s alpha for individual diagnostic scales of the Psycho-Vox questionnaire, together with discriminative power, FRES, and Jasnopis values.

Diagnostic Scale	Cronbach’s Alpha—Patients with MTD (Range of Observed Discriminatory Powers)	Cronbach’s Alpha—Control Group (Range of Observed Discriminatory Powers)	FRES/Jasnopois
Tendency to project a positive self-image	0.705 (0.25–0.65)	0.741 (0.07–0.63)	74.0/3.4
Tendency to exaggerate symptoms	0.837 (0.42–0.76)	0.850 (0.41–0.82)	36.0/5.1
Mental well-being	0.793 (0.07–0.64)	0.830 (0.07–0.75)	50.0/4.0
Perseverance	0.736 (0.13–0.64)	0.814 (0.22–0.74)	38.5/4.3
Task-oriented coping with stress	0.764 (0.27–0.68)	0.808 (0.39–0.70)	28.2/5.6
Avoiding-oriented coping with stress	0.736 (0.15–0.53)	0.838 (0.44–0.69)	45.0/4.5
Resilience	0.747 (0.34–0.51)	0.710 (0.21–0.52)	33.8/5.3
Social capital—relationships with others	0.793 (0.23–0.67)	0.659 (0.10–0.58)	63.5/3.1
Family relationships	0.822 (0.35–0.74)	0.712 (0.20–0.74)	58.9/3.4
Burnout	0.779 (0.25–0.66)	0.893 (0.47–0.82)	32.7/5.2

**Table 4:** Descriptive statistics of the Psycho-Vox indicators.

	Min	Max	M	SD	Skewness	Kurtosis
Tendency to project a positive self-image	14	40	27.80	5.01	−0.191	−0.084
Tendency to exaggerate symptoms	10	36	21.42	6.51	0.227	−0.886
Mental well-being	10	40	27.14	5.95	−0.478	−0.031
Perseverance	9	38	25.66	5.5	−0.155	−0.478
Task-oriented coping with stress	17	40	30.58	4.82	−0.585	0.183
Avoiding-oriented coping with stress	10	39	23.68	5.64	0.085	−0.054
Resilience	19	40	30.72	4.51	−0.286	−0.036
Social capital—relationships with others	19	40	30.99	4.84	−0.549	−0.195
Family relationships	13	40	28.77	5.87	−0.471	−0.177
Burnout	8	40	20.53	6.77	0.253	−0.358

M—mean; SD—standard deviation; Min—minimum value; Max—maximum value.

**Table 5:** Relationship between each group (control v. patients) and results of the Psycho-vox questionnaire based on *t*-tests for independent samples.

	Control Group		Patients with MTD		Difference		
	M	SD	M	SD	*t*	*p*	d
Tendency to project a positive self-image	26.84	5.33	28.92	4.40	−2.70	0.004	0.423
Tendency to exaggerate symptoms	20.20	6.62	22.83	6.12	−2.62	0.005	0.410
Mental well-being	26.09	6.52	28.36	4.99	−2.47	0.007	0.696
Perseverance	25.67	5.95	25.64	4.97	0.03	0.488	0.005
Task-oriented coping with stress	30.95	4.87	30.14	4.77	1.07	0.143	0.168
Avoiding-oriented coping with stress	23.07	6.13	24.39	4.95	−1.51	0.067	0.544
Resilience	30.19	4.80	31.33	4.09	−1.62	0.054	0.253
Social capital—relationships with others	30.03	4.78	32.09	4.71	−2.77	0.003	0.743
Family relationships	27.24	5.46	30.54	5.88	−3.73	<0.001	0.584
Burnout	20.90	7.58	20.11	5.71	0.75	0.228	0.117

M—mean; SD—standard deviation; *t*—Student’s *t*-statistic; *p*—statistical significance; d—Cohen’s d (measure of effect size).

**Table 6:** Relationship between age and seniority and the results of the Psycho-Vox questionnaire—Pearson’s *r* correlation coefficients.

	Control Group		Patients with MTD	
	Age	Length of Employment	Age	Length of Employment
Tendency to project a positive self-image	0.060	0.050	−0.110	−0.200
Tendency to exaggerate symptoms	−0.092	−0.190	0.180	0.150
Mental well-being	0.211 *	0.214 *	0.060	0.130
Perseverance	−0.163	−0.060	0.060	−0.020
Task-oriented coping with stress	0.266 *	0.354 **	0.030	0.030
Avoiding-oriented coping with stress	−0.186	−0.190	0.070	0.000
Resilience	0.301 **	0.387 **	−0.050	0.020
Social capital—relationships with others	−0.056	0.040	−0.190	0.000
Family relationships	0.115	0.180	0.090	0.050
Burnout	−0.304 **	−0.219 *	−0.080	−0.050

** Correlation significant at the 0.01 level (two-tailed). * Correlation significant at the 0.05 level (two-tailed).

**Table 7:** Non-standardised estimates for logistic regression.

	B	SE	Wald	*p*	Exp(B)
Tendency to project a positive self-image	0.191	0.056	11.485	<0.001	1.211
Tendency to exaggerate symptoms	0.159	0.043	13.888	<0.001	1.172
Mental well-being	0.179	0.061	8.726	0.003	1.196
Perseverance	−0.140	0.057	6.068	0.014	0.870
Task-oriented coping with stress	−0.224	0.066	11.552	<0.001	0.799
Avoiding-oriented coping with stress	0.032	0.047	0.459	0.498	1.032
Resilience	0.127	0.068	3.498	0.061	1.135
Social capital—relationships with others	0.043	0.054	0.623	0.430	1.044
Family relationships	0.093	0.042	4.909	0.027	1.097
Burnout	0.078	0.047	2.696	0.101	1.081
Constant	−13.539	3.254	17.315	<0.001	0.000

B—non-standardised estimate, *p*—significance level; Exp(B)—odds ratio.

**Table 8:** Normative values of the Psycho-Vox questionnaire for 5 scales: Tendency to project a positive self-image, Tendency to exaggerate symptoms, Mental well-being, Perseverance, and Task-oriented coping with stress.

Sten	Tendency to Project a Positive Self-Image	Tendency to Exaggerate Symptoms	Mental Well-Being	Perseverance	Task-Oriented Coping with Stress
1	below 14	below 11	below 14	below 16	below 19
2	14–19	11.12	14–16	16–17	19–22
3	20–22	13–14	17–20	18–19	23–25
4	23–25	15–17	21–24	20–22	26–29
5	26–28	18–20	25–27	23–26	30–31
6	29–30	21–25	28–30	27–28	32–33
7	31–32	26–28	31–33	29–30	34–35
8	33–35	29–31	34–35	31–33	36–37
9	36–37	32–34	36	34–35	38
10	above 37	above 34	above 36	above 35	above 38

**Table 9:** Normative values of the Psycho-Vox questionnaire for 5 scales: Avoiding-oriented coping with stress, Resilience, Social capital—relationships with others, Family relationships, and Burnout.

Sten	Avoiding-Oriented Coping with Stress	Resilience	Social Capital—Relationships with Others	Family Relationships	Burnout
1	below 12	below 21	below 20	below 16	below 9
2	12–15	21–23	20–22	16–18	9–10
3	16–17	24–26	23–26	19–23	11–13
4	18–21	27–28	27–29	24–26	14–16
5	22–23	29–30	30–31	27–29	17–20
6	24–26	31–33	32–33	30–32	21–23
7	27–29	34	34–35	33–34	24–27
8	30–32	35–37	36–37	35–37	28–30
9	33–34	38–39	38–39	38	31–35
10	above 34	40	above 39	above 38	above 35

## Data Availability

The data presented in this study are available from the corresponding author upon reasonable request.

## Supplementary Materials

The following supporting information can be downloaded at: https://www.mdpi.com/article/10.3390/jcm15114145/s1, Table S1: PSYCHO-VOX polish version; Table S2: PSYCHO-VOX english version.
