# Supplementary material for: Psycho-Vox: A Polish Questionnaire for the Differential Diagnosis of Muscle Tension Dysphonia
Source: J Clin Med. 2026 May 27;15(11):4145. doi: 10.3390/jcm15114145 (PMC13257920; doi:10.3390/jcm15114145)
Supplement: Supplementary file 1 [file jcm-15-04145-s001.zip › Psycho-Vox pl.pdf]

Table S1 PSYCHO-VOX polish version
PSYCHOVOX

Imię i nazwisko: ………………………………..

Data urodzenia: ……………………………....

Data: ……………………………………………

Instrukcja:
Poniżej znajdują się twierdzenia dotyczące tego, w jaki sposób myślą i zachowują się ludzie. Przeczytaj uważnie każde stwierdzenie i zaznacz, na ile poprawnie dana odpowiedź opisuje Ciebie. Wybraną przez Ciebie odpowiedź zaznacz przekreślając daną cyfrę w wierszu lub zakreślając ją. W kwestionariuszu nie ma dobrych ani złych odpowiedzi - ważnym jest to, abyś opisał(a) siebie takim jakim jesteś a nie takim, jakim chciał(a)byś być. Oceny dokonaj na 5- stopniowej skali:

1- zdecydowanie nie, 2 – raczej nie, 3 – trudno ocenić, 4 – raczej tak, 5 – zdecydowanie tak

**1**	Czuję, że mam siłę, aby dążyć do celu, nawet gdy pojawiają się przeszkody.	1	2	3	4	5
**2**	Mam silną emocjonalną więź z większością członków mojej rodziny.	1	2	3	4	5
**3**	Często czuję się szczęśliwy/a i zrelaksowany/a.	1	2	3	4	5
**4**	Rozważam różne opcje działania, zanim podejmę decyzję w stresującej sytuacji.	1	2	3	4	5
**5**	Mam osoby, które potrafią doradzić mi w ważnych życiowych decyzjach.	1	2	3	4	5
**6**	Jest dla mnie ważne, aby ludzie mieli o mnie dobrą opinię.	1	2	3	4	5
**7**	Gdy byłem/am dzieckiem oceniam, że miałem(am) bardzo silną więź z ojcem.	1	2	3	4	5
**8**	Skupiam się na przyjemnych czynnościach, aby zapomnieć o stresie.	1	2	3	4	5
**9**	Mam poczucie, że nie jestem w stanie odpocząć, nawet po dłuższym czasie wolnym od pracy.	1	2	3	4	5
**10**	Unikam podejmowania ryzyka w sytuacjach, które wywołują stres.	1	2	3	4	5
**11**	W naszej rodzinie czasami trudno jest dojść do porozumienia.	1	2	3	4	5
**12**	Potrafię wyciągać wnioski z porażek i wykorzystywać je w przyszłości.	1	2	3	4	5
**13**	Czuję silny niepokój, gdy zauważę u siebie jakiekolwiek nietypowe objawy zdrowotne.	1	2	3	4	5
**14**	Kiedy coś mnie stresuje, staram się odwrócić uwagę od problemu.	1	2	3	4	5
**15**	Czuję się wdzięczny/a za to, co mam.	1	2	3	4	5
**16**	Często wracam myślami do tych samych problemów, nawet jeśli nie mogę ich rozwiązać.	1	2	3	4	5
**17**	Często czuję się spięty/a i zdenerwowany/a.	1	2	3	4	5
**18**	Kiedy jestem zestresowany/a, szukam informacji, które pomogą mi zrozumieć sytuację.	1	2	3	4	5
**19**	Zdarza się, że zbyt długo trzymam się planów, które już nie mają sensu.	1	2	3	4	5
**20**	Czuję, że moi bliscy są ze mną w ważnych momentach mojego życia.	1	2	3	4	5
**21**	Wierzę, że moje działania mogą wpłynąć na poprawę trudnej sytuacji.	1	2	3	4	5
**22**	W mojej rodzinie możemy otwarcie rozmawiać o trudnych tematach.	1	2	3	4	5
**23**	Mam wrażenie, że jestem otoczony/a przez osoby, które się o mnie troszczą.	1	2	3	4	5
**24**	Czuję, że nie dbam już tak bardzo o jakość swojej pracy jak kiedyś.	1	2	3	4	5
**25**	Gdy coś mnie zdenerwuje, ten stan utrzymuje się we mnie jeszcze przez wiele godzin lub dni.	1	2	3	4	5
**26**	Czuję się pełen/a energii i gotowy/a do działania.	1	2	3	4	5
**27**	Mam tendencję do analizowania porażek przez długi czas.	1	2	3	4	5
**28**	Robię wszystko, aby odciągnąć swoje myśli od stresujących sytuacji.	1	2	3	4	5
**29**	Zawsze mówię prawdę.	1	2	3	4	5
**30**	Często rezygnuję z działania, jeśli problem wydaje się zbyt trudny.	1	2	3	4	5
**31**	Mam wrażenie, że mogę zaufać sobie w trudnych sytuacjach.	1	2	3	4	5
**32**	Starannie omijam sytuacje, które mogłyby przypomnieć mi o stresującym wydarzeniu.	1	2	3	4	5
**33**	Mam wrażenie, że inni nie rozumieją, jak bardzo moje dolegliwości zdrowotne są dla mnie uciążliwe.	1	2	3	4	5
**34**	Dbam o to, aby moje działania zawsze były postrzegane w pozytywnym świetle.	1	2	3	4	5

1- zdecydowanie nie, 2 – raczej nie, 3 – trudno ocenić, 4 – raczej tak, 5 – zdecydowanie tak

**35**	Swoje rodzinne relacje oceniam jako bardzo dobre.	1	2	3	4	5
**36**	Przez większość dnia mam obniżony nastrój.	1	2	3	4	5
**37**	Zwracam dużą uwagę na dolegliwości zdrowotne w moim ciele.	1	2	3	4	5
**38**	Myśl o kolejnym dniu pracy wywołuje we mnie zniechęcenie.	1	2	3	4	5
**39**	Mam wokół siebie ludzi, którzy pomagają mi lepiej zrozumieć różne sytuacje.	1	2	3	4	5
**40**	Często stresuję się przez mój stan zdrowia.	1	2	3	4	5
**41**	Czuję, że moje doświadczenia uczyniły mnie bardziej odpornym/ą na przyszłe trudności.	1	2	3	4	5
**42**	W mojej rodzinie są osoby, które mają ze sobą konflikt.	1	2	3	4	5
**43**	Wiem, że mogę liczyć na wsparcie grupy przyjaciół lub znajomych.	1	2	3	4	5
**44**	Czuję się wyczerpany/a emocjonalnie po pracy.	1	2	3	4	5
**45**	Mam skłonność do zamartwiania się błędami, które popełniłem/am w przeszłości.	1	2	3	4	5
**46**	Czuję niepokój, gdy coś zostaje niedokończone.	1	2	3	4	5
**47**	Wiem, że mogę liczyć na pomoc innych w codziennych obowiązkach, gdy tego potrzebuję.	1	2	3	4	5
**48**	Unikam kontaktów z innymi w pracy, gdy tylko mam taką możliwość.	1	2	3	4	5
**49**	W sytuacjach stresowych szukam konkretnego rozwiązania problemu.	1	2	3	4	5
**50**	Często zastanawiam się, jak moje działania są postrzegane przez innych.	1	2	3	4	5
**51**	Czuję się ciągle zmęczony bez wyraźnego powodu.	1	2	3	4	5
**52**	Tworzę listę rzeczy do zrobienia, aby uporządkować swoje działania w trudnych sytuacjach.	1	2	3	4	5
**53**	Czuję się niespokojny/a, dopóki nie upewnię się, że moje dolegliwości zdrowotne są niegroźne.	1	2	3	4	5
**54**	Odczuwam wahania nastroju bez wyraźnego powodu.	1	2	3	4	5
**55**	Staram się jak najszybciej rozwiązać problem, który wywołuje stres.	1	2	3	4	5
**56**	Często widzę pozytywne strony nawet w trudnych okolicznościach.	1	2	3	4	5
**57**	Potrafię radzić sobie z sytuacjami, które wymagają szybkiego podejmowania decyzji.	1	2	3	4	5
**58**	Często planuję, co powiedzieć lub zrobić, aby dobrze wypaść w oczach innych.	1	2	3	4	5
**59**	Często czuję złość, gdy inni bagatelizują moje dolegliwości zdrowotne.	1	2	3	4	5
**60**	Zdarzyło mi się oszukać kogoś (np. w grze).	1	2	3	4	5
**61**	Szybko dostosowuję się do zmian w otoczeniu.	1	2	3	4	5
**62**	Gdy jestem w coś zaangażowany/a, trudno mi się oderwać, nawet na chwilę.	1	2	3	4	5
**63**	Często staram się zapomnieć o problemach, które mnie niepokoją.	1	2	3	4	5
**64**	Czuję dyskomfort, gdy ktoś zauważy moje błędy lub niedociągnięcia.	1	2	3	4	5
**65**	Czuję, że mam silne więzi społeczne z osobami w moim otoczeniu.	1	2	3	4	5
**66**	Inni zwracają mi uwagę, że za bardzo przejmuję się swoim zdrowiem.	1	2	3	4	5
**67**	Jeśli coś nie idzie po mojej myśli, trudno mi zmienić kierunek działania.	1	2	3	4	5
**68**	Wolę unikać trudnych rozmów lub działań, gdy sytuacja mnie stresuje.	1	2	3	4	5
**69**	Traktuję swoją pracę bardziej jako przymus niż jako coś wartościowego.	1	2	3	4	5
**70**	Mam wrażenie, że moje dolegliwości zdrowotne są bardziej dotkliwe, niż to się wydaje innym.	1	2	3	4	5
**71**	Korzystam z doświadczeń z przeszłości, aby znaleźć najlepsze rozwiązanie problemu.	1	2	3	4	5
**72**	Mam poczucie równowagi emocjonalnej w większości dni.	1	2	3	4	5
**73**	W sytuacji stresowej koncentruję się na rozwiązywaniu problemu, zamiast na emocjach.	1	2	3	4	5
**74**	Gdy byłem/am dzieckiem oceniam, że miałem(am) bardzo silną więź z matką.	1	2	3	4	5
**75**	Czuję się zmęczony/a, nawet gdy dopiero zaczynam dzień pracy.	1	2	3	4	5
**76**	Staram się unikać zachowań, które mogłyby sprawić, że inni pomyślą o mnie źle.	1	2	3	4	5
**77**	Czuję, że mam osoby, które potrafią mnie wysłuchać, gdy tego potrzebuję.	1	2	3	4	5
**78**	Czuję, że nie osiągam nic wartościowego w swojej pracy.	1	2	3	4	5
**79**	Ustalam priorytety, aby skupić się na najważniejszych działaniach w obliczu stresu.	1	2	3	4	5
**80**	Mam wokół siebie osoby, z którymi mogę dzielić swoje radości i sukcesy.	1	2	3	4	5

Sprawdź, czy udzieliłeś(aś) odpowiedzi na wszystkie pytania. Dziękujemy!
