# Supplementary material for: Psycho-Vox: A Polish Questionnaire for the Differential Diagnosis of Muscle Tension Dysphonia
Source: J Clin Med. 2026 May 27;15(11):4145. doi: 10.3390/jcm15114145 (PMC13257920; doi:10.3390/jcm15114145)
Supplement: Supplementary file 1 [file jcm-15-04145-s001.zip › Psycho-Vox 22 3 eng kwestionariusz suppl file-AB.pdf]

Table S2: PSYCHO-VOX english version
PSYCHO-VOX

Name: ....   Date of birth:.......................................

Instructions:
Below is a list of numbered statements about how some people think and behave. Read each statement carefully and assess how correctly the answer describes you. Mark the answer you by putting a cross through the relevant number on the right. There are no right or wrong answers – the important thing is that you describe yourself as you are, not as you would like to be. Please rate the statements on a 5-point scale, where:

1 – definitely not, 2 – probably not, 3 – hard to say, 4 – mostly yes, 5 – definitely yes

**1**	I feel I have the strength to pursue my goal, even when obstacles arise.	1	2	3	4	5
**2**	I have a strong emotional bond with most of my family members.	1	2	3	4	5
**3**	I often feel happy and relaxed.	1	2	3	4	5
**4**	In a stressful situation I consider different options for action before making a decision.	1	2	3	4	5
**5**	I have people who can advise me on important life decisions.	1	2	3	4	5
**6**	It's important to me that people have a good opinion of me.	1	2	3	4	5
**7**	When I was a child, I had a very strong bond with my father.	1	2	3	4	5
**8**	To forget about stress I focus on pleasurable activities.	1	2	3	4	5
**9**	I feel like I'm not able to relax, even after a long time away from work.	1	2	3	4	5
**10**	I avoid taking risks in situations that cause stress.	1	2	3	4	5
**11**	In our family, it is sometimes difficult to come to an agreement.	1	2	3	4	5
**12**	I can learn from failures and use them in the future.	1	2	3	4	5
**13**	I feel very anxious when I notice any unusual health symptoms in myself.	1	2	3	4	5
**14**	When something stresses me out, I try to distract myself from the problem.	1	2	3	4	5
**15**	I feel grateful for what I have.	1	2	3	4	5
**16**	I often keep thinkingback to the same problems, even if I can't solve them.	1	2	3	4	5
**17**	I often feel tense and nervous.	1	2	3	4	5
**18**	When I'm stressed, I look for information that helps me understand the situation.	1	2	3	4	5
**19**	I stick to plans for too long even if they no longer make sense.	1	2	3	4	5
**20**	I feel that my loved ones are with me in important moments of my life.	1	2	3	4	5
**21**	I believe that my actions can improve a difficult situation.	1	2	3	4	5
**22**	In my family, we can talk openly about difficult topics.	1	2	3	4	5
**23**	I feel like I'm surrounded by people who care about me.	1	2	3	4	5
**24**	I feel I don't care as much as I used to about the quality of my work.	1	2	3	4	5
**25**	If something makes me nervous, I stay this way for many hours or days.	1	2	3	4	5
**26**	I feel energized and ready to act.	1	2	3	4	5
**27**	I tend to analyze failures for a long time.	1	2	3	4	5
**28**	I do everything to take my mind off stressful situations.	1	2	3	4	5
**29**	I always tell the truth.	1	2	3	4	5
**30**	I often give up if the problem seems too difficult.	1	2	3	4	5
**31**	I feel like I can trust myself in difficult situations.	1	2	3	4	5
**32**	I carefully avoid situations that remind me of a stressful event.	1	2	3	4	5
**33**	I feel that others don't understand how much my health ailments bother me.	1	2	3	4	5
**34**	I make sure that my actions are always perceived in a positive light.	1	2	3	4	5

1 – definitely not, 2 – probably not, 3 – hard to say, 4 – mostly yes, 5 – definitely yes

**35**	I rate my family relationships as very good.	1	2	3	4	5
**36**	I am in a low mood for most of the day.	1	2	3	4	5
**37**	I pay a lot of attention to my body's health ailments.	1	2	3	4	5
**38**	The thought of another day of work makes me feel discouraged.	1	2	3	4	5
**39**	I have people around me who help me better understand different situations.	1	2	3	4	5
**40**	I often get stressed about my health.	1	2	3	4	5
**41**	I feel my experiences have made me more resilient to future difficulties.	1	2	3	4	5
**42**	There are people in my family who have conflicts between themselves.	1	2	3	4	5
**43**	I know I can count on the support of a group of friends or acquaintances.	1	2	3	4	5
**44**	I feel emotionally drained after work.	1	2	3	4	5
**45**	I tend to worry about mistakes I've made in the past.	1	2	3	4	5
**46**	I feel anxious when something is left unfinished.	1	2	3	4	5
**47**	I know that I can count on the help of others in my daily tasks when I need it.	1	2	3	4	5
**48**	I avoid contact with others at work whenever I can.	1	2	3	4	5
**49**	In stressful situations, I look for a specific solution to the problem.	1	2	3	4	5
**50**	I often wonder how my actions are perceived by others.	1	2	3	4	5
**51**	I feel tired all the time for no apparent reason.	1	2	3	4	5
**52**	In a difficult situation I create a to-do list to organize my actions.	1	2	3	4	5
**53**	I feel anxious until I'm sure my health conditions are harmless.	1	2	3	4	5
**54**	I experience mood swings for no apparent reason.	1	2	3	4	5
**55**	I try to solve a problem that causes stress as soon as possible.	1	2	3	4	5
**56**	I see the positive side of things even in a difficult circumstance.	1	2	3	4	5
**57**	I can deal with situations that require quick decision-making.	1	2	3	4	5
**58**	I plan what to say or do in order to look good in the eyes of others.	1	2	3	4	5
**59**	I feel angry when others downplay my health ailments.	1	2	3	4	5
**60**	I am readily able to deceive someone (e.g. in a game).	1	2	3	4	5
**61**	I quickly adapt to changes in the environment.	1	2	3	4	5
**62**	When I'm involved in something, it's hard for me to tear myself away, even for a moment.	1	2	3	4	5
**63**	I often try to forget about the problems that bother me.	1	2	3	4	5
**64**	I feel uncomfortable when someone notices my mistakes or shortcomings.	1	2	3	4	5
**65**	I feel I have strong social ties with those around me.	1	2	3	4	5
**66**	Others point out to me that I worry too much about my health.	1	2	3	4	5
**67**	If something doesn't go my way, it's hard for me to change the course of action.	1	2	3	4	5
**68**	I prefer to avoid difficult conversations or actions when the situation is stressful for me.	1	2	3	4	5
**69**	I treat my work more as a compulsion than as something valuable.	1	2	3	4	5
**70**	I have the impression that my health ailments are more severe than others think.	1	2	3	4	5
**71**	I use past experience to find the best solution to a problem.	1	2	3	4	5
**72**	On most days I have a sense of emotional balance.	1	2	3	4	5
**73**	In a stressful situation, I focus on solving the problem instead of the surrounding emotions.	1	2	3	4	5
**74**	When I was a child, I had a very strong bond with my mother.	1	2	3	4	5
**75**	I feel tired, even when I'm just starting my workday.	1	2	3	4	5
**76**	I try to avoid behaviors that could make others think badly of me.	1	2	3	4	5
**77**	I feel I have people who can listen to me when I need it.	1	2	3	4	5
**78**	I feel I am not achieving anything of value in my work.	1	2	3	4	5
**79**	In the face of stress I set priorities to focus on what is most important to do.	1	2	3	4	5
**80**	I have people around me with whom I can share my joys and successes.	1	2	3	4	5

Check that you've answered all your questions. Thank you!
